# Association Between Circulating Lymphocyte Populations and Outcome After Stereotactic Body Radiation Therapy in Patients With Hepatocellular Carcinoma

**DOI:** 10.3389/fonc.2019.00896

**Published:** 2019-09-10

**Authors:** Yuan Zhuang, Bao-ying Yuan, Gen-wen Chen, Xiao-mei Zhao, Yong Hu, Wen-chao Zhu, Zhao-chong Zeng, Yi-xing Chen

**Affiliations:** Department of Radiation Oncology, Zhongshan Hospital, Fudan University, Shanghai, China

**Keywords:** stereotactic body radiation therapy, hepatocellular carcinoma, overall survival, lymphopenia, circulating lymphocyte populations

## Abstract

**Background and Objective:** Radiation-induced lymphopenia has a tangible impact on overall survival (OS) in multiple solid tumors. We investigated the association between circulating lymphocyte populations (CLPs) before and after stereotactic body radiation therapy (SBRT) and OS in patients with hepatocellular carcinoma (HCC).

**Materials and Methods:** Seventy-eight HCC patients treated with SBRT between January 2013 and June 2017 were retrospectively analyzed. Baseline and post-treatment total peripheral lymphocyte counts (TPLCs) and values of different CLPs were obtained and analyzed for clinical outcomes. Univariate and multivariate Cox regression analyses were used to explore the independent prognostic factors for patient survival.

**Results:** The one-, two- and three-year OS rates were 94.8, 75.9, and 63.3%, respectively. The mean TPLCs before and 10 days after SBRT were 1.4 × 10^9^/L and 0.7 × 10^9^/L, respectively. The TPLC recovered to its baseline value 1 year after SBRT. Multivariate analysis results revealed that variables, including tumor necrosis factor-alpha (TNF-α) level <5.5 ng/mL and post-treatment TPLC <0.45 × 10^9^/L were independent factors for inferior OS. Further analysis showed that the values of CLPs, including CD3^+^, CD4^+^, CD8^+^, CD19^+^, and CD16^+^56^+^ cells dropped profoundly 10 days after SBRT, among which CD19^+^ B cell count was mostly depleted and gradually recovered after 2 months. Univariate analysis showed that both baseline and post-treatment TPLC and CLP (except post-treatment B cell) counts were significantly associated with patient OS (*p* < 0.05 for each). Further stratified analysis performed according to OS at 2 years demonstrated that the CD16^+^CD56^+^ NK cell counts remained significantly elevated in patients with better survival (OS > 2 years) compared to those in short-term survivors at 10 days, 1 month, and 2 months after SBRT (*p* < 0.05 for each). In addition, there were significant differences in TPLC and CD8^+^ T cell counts in patients with long-term and short-term OS at 2 months after SBRT (*p* < 0.05).

**Conclusions:** Peripheral lymphopenia after SBRT might be an independent prognostic factor for poorer outcome in HCC patients. Post-treatment lymphocyte subsets, including CD8^+^ T cell and NK cell counts were also associated with 2-year OS rates.

## Introduction

Hepatocellular carcinoma (HCC) is the most common primary malignancy of the liver, representing the third-leading cause of cancer mortality worldwide (1). With the progression of diagnostic imaging, small-sized HCC are now more likely to be detected with high accuracy. Of the current therapeutic approaches for small HCC, surgical management is the standard treatment for patients with well-preserved liver function (2). However, only a minority of patients are candidates for curative surgical treatment at the time of diagnosis (3). Stereotactic body radiation therapy (SBRT) has emerged as a safe and effective treatment option for patients with inoperable HCC and is significantly correlated with favorable local control rates and survival outcomes (4). Several clinical trials examining SBRT use have reported 3-year overall survival (OS) and local control rates of 54.0–78.6 and 89.3–100%, respectively, in small HCC (5–9).

However, radiotherapy (RT) for peripheral organs, particularly when delivered over protracted courses, can cause rapid depletion of total peripheral lymphocyte count (TPLC) (10, 11). Moreover, these responses may vary between individuals and different circulation lymphocyte population (CLP) types (12–14). Nowadays, the recognition that the immune system plays a vital role in tumor surveillance and the advent of immunotherapy has renewed the focus on preserving a pool of functioning lymphocytes in circulation. RT-induced lymphopenia (RIL) has been associated with poor outcome in liver cancer (11, 15), breast cancer (16), glioblastoma (17), nasopharyngeal cancer (18), non-small cell lung cancer (NSLC) (19), pancreatic cancer (20), and other tumors (21–23). Nevertheless, few reports have focused on the impact of SBRT on the values of different CLP types, including CD3^+^ T, CD19^+^ B, and CD15^+^56^+^ NK cells and the prognostic values of CLPs for survival in HCC patients. To our knowledge, our study is the first to examine the association between different CLPs both before and after SBRT with the clinical outcome of patients with HCC.

## Materials and Methods

### Patient Selection

We retrospectively examined the medical records of patients with HCC who were not suitable for surgery between January 2013 and June 2017. The inclusion criteria for patients receiving SBRT were as follows: (1) HCC confirmed by histologic or imaging criteria based on the National Comprehensive Cancer Network guidelines for hepatobiliary cancers, (2) age > 18 years, (3) Child-Pugh class A or B, Eastern Co-operative Oncology Group performance status 0 or 1 and the Barcelona Clinic Liver Cancer 0-A stages within 1 month before SBRT, (4) laboratory tests taken within 1 month before and 1 month after SBRT, and (5) one or more radiological evaluations before and after SBRT. Patients with any of the following conditions were excluded from the study: (1) distant metastasis, (2) double primary malignancy, or (3) no follow-up or follow-up lasting <6 months after the completion of SBRT. This research was approved by the Institutional Review Board of the Ethics Committee at our institution and performed in accordance with the principles of the Declaration of Helsinki.

### SBRT

All enrolled patients were trained to maintain slow breathing with respiratory exercises before the implementation of SBRT. The Body Pro-Lok system was used for abdominal compression to reduce the amplitude of liver motion. Patients underwent a contrast-enhanced computed tomography (CT) simulation while immobilized by a customized vacuum body mold in the supine position. The gross tumor volume included all tumors detected via dynamic CT. For tumors that were not well visualized by CT scan, a pre-treatment magnetic resonance imaging (MRI) study was registered to the planning CT. Four-dimensional CT simulations were used to generate an internal target volume (ITV). The planning target volume (PTV) was defined as the ITV plus a radial margin of 3 mm. SBRT was planned using the TomoTherapy Planning System (Accuray, Inc., Madison, WI). A radiotherapy dose was prescribed to the isodose surface covering 95% of the PTV. Patients received 5–10 fractions, delivered five times per week with a median dose of 48 Gray (Gy) and a range of 48–60 Gy (50 Gy/5 fractions for 13 patients, 50–60 Gy/10 fractions for 8 patients, and 48–54 Gy/6 fractions for 57 patients).

### Follow-Up and Patient Outcomes

Follow-up was defined from the start date of SBRT to determine the median follow-up and time-to-event estimates. To assess the impact of SBRT on blood volume, baseline peripheral blood cell counts, including white blood cells (WBCs), platelets (PLTs), red blood cells (RBCs), and CLPs within 3 days prior to SBRT were obtained, while post-treatment values were analyzed at 10 days; 1, 2, and 3 months after SBRT; and every 3 months thereafter. If peripheral cell records were not available at a certain month, the closest values to the month were used. Follow-up CT or MRI studies were performed 6–8 weeks after SBRT and tri-monthly thereafter. The recurrence of tumor, an increase in the size of the primary tumor, or the development of regional or distant metastasis was defined as progression. OS was calculated at the patient level as the time from the first SBRT until death from any cause or the last follow-up. Progression-free survival (PFS) was calculated as the interval between the first SBRT and disease progression, last follow-up, or death. Local control was defined as freedom from local progression according to modified Response Evaluation Criteria in Solid Tumors (mRECIST) guidelines.

### Statistics and Analysis

Descriptive statistics were summarized as means ± standard deviation or as medians and interquartile range, depending on whether the data were distributed normally according to Kolmogorov–Smirnov tests. Comparisons between quantitative variables were estimated by two-sided t- or Mann–Whitney tests, as appropriate. The primary and secondary endpoints were OS and PFS, respectively. Cumulative survival was calculated using the Kaplan–Meier method and the cutoff values of the continuous variables for patient prognosis were determined by maximally selected log-rank tests (24). Univariate and multivariate analyses were performed using Cox and logistic regression models with hazard ratios (HRs) and 95% confidence intervals (95% CIs). Multivariate analysis was performed using the statistically significant factors identified in univariate analysis (*p* < 0.05). Data were analyzed using IBM SPSS Statistics for Windows, version 23.0 (IBM Corp., Armonk, NY, USA).

## Results

### Patient Characteristics and Clinical Outcomes

Between January 2013 to June 2017, a total of 78 patients with small HCC were included and fully evaluated in the present study. Their baseline characteristics are summarized in Table 1. The mean age was 59.4 ± 13.3 years and the median tumor size was 2.5 cm. Among all patients, seventy-five patients (96.2%) had Child-Pugh grade A disease while three patients had Child-Pugh B grade within 1 month before SBRT. The median follow-up duration was 32.0 (range: 4.1–80.0) months. At the time of our analysis, the 1-, 2- and 3-year OS rates were 94.8, 75.9, and 63.3%, respectively. The one-, two- and three-year PFS rates were 76.7, 55.0, and 42.1%, respectively. The median PFS was 21.4 (range: 1.8–66.9) months. In addition, the 1-, 2- and 3-year local control rates after SBRT were 96.1, 92.3, and 89.7%, respectively.

**Table 1:** Patient baseline demographics.

**Characteristics**	**Patients (*n* = 78)**	**Percentage (%)**
Age (years, mean ± SD)	59.4 ± 13.3	
Sex		
Male/female	64/14	82.1/17.9
Median tumor size (cm)	2.5 (1.5–3.0)	
Gross tumor volume (cm^3^)	15.0 (6.5–38.1)	
Plan tumor volume (cm^3^)	43.0 (25.8–83.7)	
Biologically effective dose (Gy)	96.0 (86.4–100.0)	
Red blood cell counts (× 10^12^/L)	4.6 ± 0.6	
White blood cell count (× 10^9^/L)	5.4 ± 1.8	
Platelet count (×10^9^/L)	128.2 ± 57.8	
TPLC (×10^9^/L)	1.4 ± 0.6	
Alpha-fetoprotein (ng/mL)	1028.1 ± 2370.8	
TNF-α (ng/mL)	8.5 ± 4.4	
Child-Pugh classification		
Child A/Child B	75/3	96.2/3.8
Presence of branch portal vein involvement	3	3.8
Viral etiology		
Hepatitis B	63	80.8
Hepatitis C	2	2.6
None	13	16.7
Previous treatments	57	73.1
No	21	26.9
Surgery treatment	28	35.9
TACE or RFA or PEI	40	51.3
No. of lesions		
1	74	94.9
2	4	5.1

*TPLC, total peripheral lymphocyte counts; TNF-α, tumor necrosis factor-α; TACE, transarterial chemoembolization; RFA, radiofrequency ablation; PEI, percutaneous ethanol injection. Unless otherwise noted, data are numbers with percentages, or medians with interquartile ranges in parentheses or presented as mean ± standard deviation as appropriate*.

### Impact of SBRT on Peripheral Blood Cells and Factors Associated With RIL

**Figure 1:**
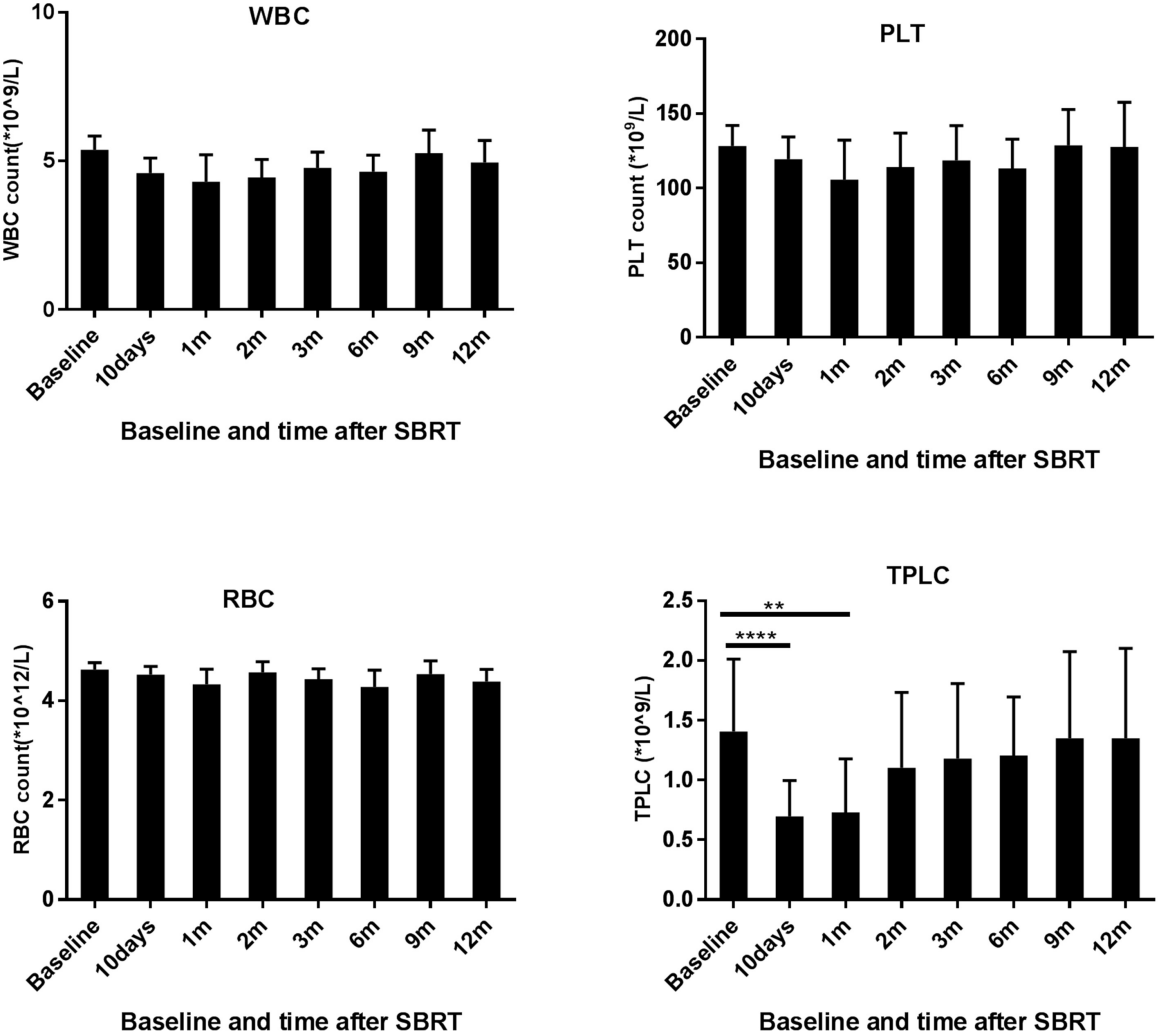
Effects of stereotactic body radiation therapy (SBRT) on peripheral blood cells in 78 patients with hepatocellular carcinoma. The counts of white blood cells (WBCs), red blood cells (RBCs), and platelets (PLTs) and total peripheral lymphocyte count (TPLC) were calculated at baseline (within 3 days prior to SBRT) and at different time points since SBRT (10 days and 1, 2, 3, 6, 9, and 12 months) as indicated for patients with HCC. **Significantly different at *p* < 0.01 compared to the baseline values. ****Significantly different at *p* < 0.0001 compared to the baseline values.

**Figure 2:**
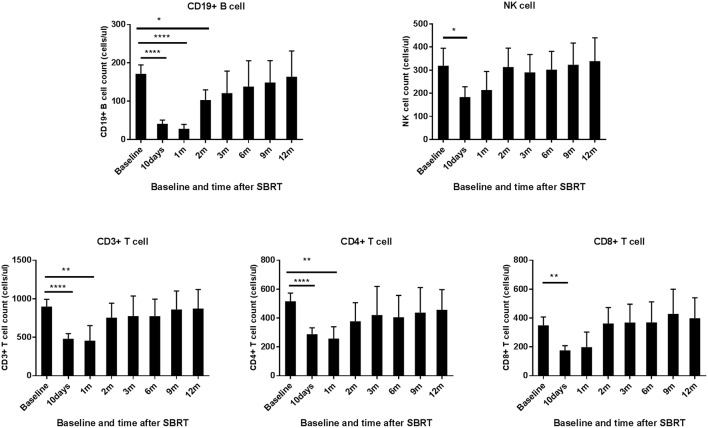
Effects of stereotactic body radiation therapy (SBRT) on circulating lymphocyte population cells in patients with hepatocellular carcinoma (HCC). The absolute numbers of CD 19^+^ B cell; NK cell; and CD 3^+^, CD 4^+^, and CD 8^+^ T cells were calculated at baseline (within 3 days prior to SBRT) and at different time points after SBRT (10 days and 1, 2, 3, 6, 9, and 12 months) as indicated for patients with HCC. *Significantly different at *p* < 0.05 compared to baseline values. **Significantly different at *p* < 0.01 compared to the baseline values. ****Significantly different at *p* < 0.0001 compared to the baseline values.

When compared to the baseline levels, the counts of WBCs, PLTs, and RBCs showed no significant changes after SBRT (Figure 1). However, TPLC was significantly decreased 10 days after SBRT and gradually increased from 2 months. The mean pre-treatment TPLC was 1.41 × 10^9^/L and decreased to 0.70 × 10^9^/L at 10 days after SBRT. Two months after SBRT, the TPLC had recovered to a mean value of 1.10 × 10^9^/L (within the normal range) and had recovered to its baseline value 1 year after SBRT (Figure 1). Further analysis was performed according to CLPs, including CD3^+^ T, CD4^+^ T, CD8^+^ T, CD19^+^ B, and CD16^+^CD56^+^ NK cells, respectively. As shown in Table 2 and Figure 2, the peripheral B cell counts were profoundly depleted at 10 days after SBRT (from 171.7 to 41.4 cells/μl) and remained low for 2 months after therapy (*p* < 0.05). Although peripheral blood CD3^+^, CD4^+^, and CD8^+^ T, and NK cell counts followed the B-lymphocyte pattern of depletion, they dropped less (nearly half after SBRT) than B cells and recovered slightly sooner. The mean values of TPLC and CLPs at different times after SBRT are listed in Table 2. In addition, logistic regression analysis was used to elucidate the factors causing severe lymphopenia 10 days after SBRT (Table 3). The results showed that a larger PTV was independently associated with an increased risk of RIL (HR: 1.44; 95% CI: 0.51–6.12; *p* < 0.05).

**Table 2:** Mean TPLCs and circulating lymphocyte population values at different times after SBRT.

**Counts of lymphocytes**	**Baseline values**	**Absolute lymphocyte counts at different times after SBRT**						
		**10 days**	**1 month**	**2 months**	**3 months**	**6 months**	**9 months**	**12 months**
TPLCs (×10^9^/L)	1.41 ± 0.61	0.70 ± 0.30	0.73 ± 0.45	1.10 ± 0.63	1.18 ± 0.63	1.21 ± 0.49	1.35 ± 0.72	1.35 ± 0.75
CD3^+^ cell (cells/μL)	897.7 ± 332.7	479.5 ± 214.8	456.3 ± 314.7	754.6 ± 444.6	787.1 ± 507.8	775.6 ± 522.7	860.7 ± 414.9	872.9 ± 425.9
CD4^+^ cell (cells/μL)	515.5 ± 202.0	287.4 ± 144.2	257.6 ± 142.6	377.0 ± 292.3	420.2 ± 359.1	405.5 ± 293.3	437.1 ± 273.2	456.0 ± 88.2
CD8^+^ cell (cells/μL)	342.5 ± 208.1	171.8 ± 106.0	184.1 ± 173.5	346.4 ± 225.6	341.7 ± 265.6	345.7 ± 265.7	389.5 ± 260.8	398.3 ± 263.7
CD19^+^ cell (cells/μL)	171.7 ± 82.0	41.4 ± 30.0	28.3 ± 18.4	103.4 ± 60.0	121.4 ± 94.8	138.4 ± 105.6	148.8 ± 107.3	163.8 ± 106.1
NK cell (cells/μL)	318.9 ± 269.8	183.6 ± 148.4	213.8 ± 189.9	313.3 ± 219.2	290.0 ± 179.8	301.7 ± 174.1	323.3 ± 169.7	338.9 ± 181.9

*TPLCs, total peripheral lymphocyte counts; SBRT, stereotactic body radiation therapy*.

**Table 3:** Factors associated with peripheral lymphopenia in patients with hepatocellular carcinoma treated with stereotactic body radiation therapy by logistic analysis.

**Variables**	**Univariate analysis**		**Multivariate analysis**	
	**HR (95% CI)**	***p-*value**	**HR (95% CI)**	***p-*value**
Age (≥60 years)	0.35 (0.09–1.28)	0.112		
Sex (female)	0.76 (0.17–3.43)	0.716		
Presence of hepatitis	1.75 (0.47–6.53)	0.043	1.96 (0.69–9.01)	0.282
Presence of previous treatments	2.46 (0.71–4.52)	0.686		
Tumor size (≥1.5 cm)	1.91 (0.46–7.93)	0.379		
Red blood cells (≥4.5 × 10^12^/L)	0.31 (0.06–1.50)	0.145		
Platelets (≥80 × 10^9^/L)	0.32 (0.08–1.23)	0.098		
TNF-α (≥6.3 ng/mL)	0.27 (0.04–1.71)	0.166		
AFP (≥25.0 ng/mL)	3.56 (0.40–31.68)	0.256		
Biologically effective dose (≥96.0 Gy)	2.63 (0.71–9.74)	0.149		
Plan tumor volume (≥83.7 cm^3^)	1.81 (0.60–6.41)	0.003	1.44 (0.51–6.12)	0.003
Baseline TPLC (≥1.45 × 10^9^/L)	0.29 (0.10–0.79)	0.198		

*TNF-α, tumor necrosis factor-α; AFP, alpha-fetoprotein; TPLC, total peripheral lymphocytes count; HR, hazard ratio; CI, confidence interval*.

### Independent Prognostic Factors for Patients Treated With SBRT

The optimal cutoff values determined by the maximally selected log-rank method were used to estimate the best prognostic cutoffs for continuous variables. Factors including age; sex; tumor size; PTV; biologically effective dose; presence or absence of chronic hepatitis; and baseline counts of RBCs, WBCs, PLTs, tumor necrosis factor-α (TNF-α) level, alpha-fetoprotein (AFP) level; and both baseline and post-treatment TPLC and CLP values at 10 days after SBRT were assessed in univariate analyses. The results revealed that tumor size ≥1.5 cm, PTV ≥83.7 cm^3^, AFP ≥25.0 ng/ml, a low level of TNF-α, WBC, baseline and post-treatment TPLC and CLP counts except for post-treatment B cell value, were significantly associated with poor OS (Table 4, *p* < 0.05 for each). After adjusting for covariates, the association of the above factors with OS was analyzed using a Cox regression model. The results revealed that TNF-α <5.5 ng/ml (HR: 0.07; 95% CI: 0.01–0.44; *p* = 0.005) and post-treatment TPLC <0.45 × 10^9^/l (HR: 0.14; 95% CI: 0.02–0.93; *p* = 0.040) were independent adverse factors for OS (Table 4).

**Table 4:** Univariate and multivariate analyses of overall survival in patients with hepatocellular carcinoma treated with stereotactic body radiation therapy.

**Variables**	**Univariate analysis**		**Multivariate analysis**	
	**HR (95% CI)**	***p-*value**	**HR (95% CI)**	***p-*value**
Age (≥60 years)	0.58 (0.26–1.31)	0.189		
Sex (female)	1.42 (0.42–4.79)	0.572		
Presence of hepatitis	2.80 (0.92–8.53)	0.070		
Biologically effective dose (≥96.0 Gy)	0.86 (0.37–1.99)	0.721		
Presence of previous treatments	2.46 (0.71–4.52)	0.155		
Tumor size (≥1.5 cm)	2.99 (1.00–8.95)	0.050	3.34 (0.29–10.97)	0.337
Red blood cells (≥4.5 × 10^12^/L)	0.41 (0.16–1.03)	0.057		
White blood cells (≥4.2 × 10^9^/L)	0.26 (0.11–0.65)	0.004		
Platelets (≥80.0 × 10^9^/L)	0.44 (0.18–1.06)	0.068		
TNF-α (≥6.3 ng/mL)	0.14 (0.05–0.43)	0.001	0.07 (0.01–0.44)	0.005
AFP (≥25.0 ng/mL)	6.00 (0.81–44.9)	0.034	2.13 (0.21–21.86)	0.524
Plan tumor volume (≥83.7 cm^3^)	4.02 (1.71–9.44)	0.001		
Baseline TPLC (≥1.45 × 10^9^/L)	0.29 (0.10–0.79)	0.044		
Baseline CD19^+^ cells (≥148.5 cells/μL)	0.36 (0.13–0.99)	0.039		
Baseline CD3^+^ T cells (≥701.0 cells/μL)	0.30 (0.11–0.80)	0.011		
Baseline CD4^+^ T cells (≥537.5 cells/μL)	0.17 (0.05–0.58)	0.002		
Baseline CD8^+^ T cells (≥356.0 cells/μL)	0.29 (0.08–1.02)	0.040		
Baseline CD56^+^ T cells (≥390.5 cells/μL)	0.20 (0.05–0.90)	0.021		
Post-treatment TPLC (≥0.45 × 10^9^/L)	0.18 (0.10–0.79)	<0.001	0.14 (0.02–0.93)	0.040
Post-treatment CD19^+^ cells (≥52.5 cells/μL)	0.55 (0.15–2.07)	0.375		
Post-treatment CD3^+^ T cells (≥353.0 cells/μL)	0.29 (0.09–0.96)	0.004		
Post-treatment CD4^+^ T cells (≥231.0 cells/μL)	0.25 (0.08–0.83)	0.006		
Post-treatment CD8^+^ T cells (≥179.0 cells/μL)	0.08 (0.01–0.64)	0.002		
Post-treatment NK cells (≥76.5 cells/μL)	0.21 (0.06–0.69)	0.002		

*TNF-α, tumor necrosis factor-α; AFP, alpha-fetoprotein; TPLC, total peripheral lymphocyte counts; HR, hazard ratio; CI, confidence interval*.

**Figure 3:**
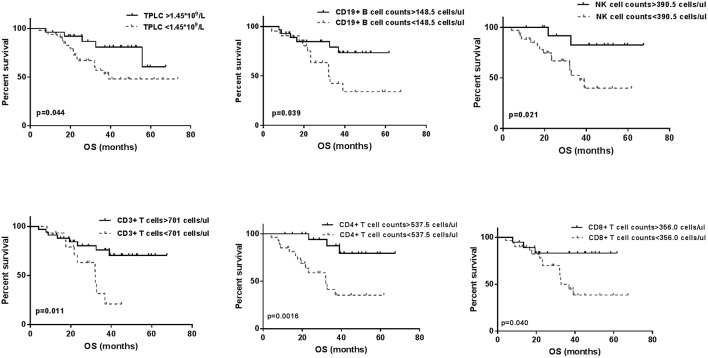
The Kaplan-Meier overall survival (OS) curves for baseline total peripheral lymphocyte count (TPLC) and different circulating lymphocyte populations (CLPs). Within 3 days prior to stereotactic body radiation therapy, significant differences for high level (—) and low level (- - -) of TPLC and all CLP types for OS curves were observed (*p* < 0.05 for each).

Regarding baseline CLPs, the cumulative survivals were calculated using the Kaplan–Meier method, respectively, as shown in Figure 3. There were significant differences in the OS curves between higher and lower CLP levels (*p* < 0.05 for each), which indicated a positive relationship between pre-treatment TPLC and different CLP types and patient OS.

### Association of CLP Depletion After SBRT With Patient Survival

**Figure 4:**
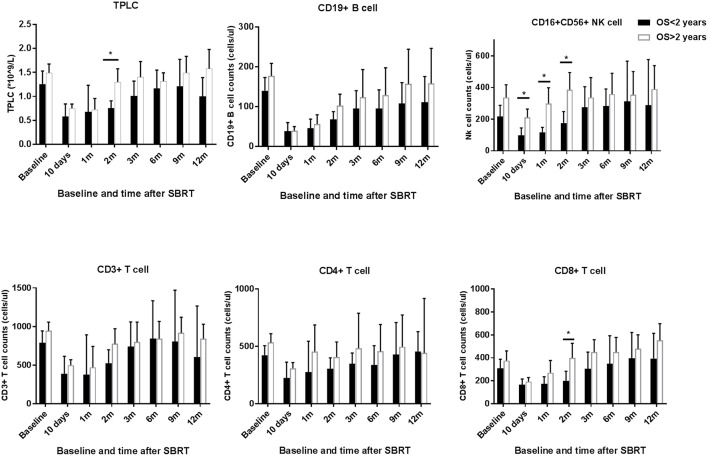
TPLC and circulating lymphocyte population (CLP) counts after stereotactic body radiation therapy (SBRT) stratified by long or short overall survival (OS > 2 years vs. OS < 2 years). The absolute numbers of TPLC and CLPs were calculated at baseline (within 3 days prior to SBRT) and at different time points after SBRT (10 days and 1, 2, 3, 6, 9, and 12 months) as indicated for hepatocellular carcinoma patients. *Significantly different at *p* < 0.05 compared to the baseline values.

To assess whether the relationship between post-treatment CLPs and patient survival after SBRT, we stratified patients according to OS at 2 years after therapy. As shown in Figure 4, patients with an inferior OS (OS <2 years) tended to have lower CLP counts compared to those in patients with long-term survival (OS > 2 years). The peripheral blood NK cell counts of patients with better survival remained significantly elevated compared to those of short-term survivors at 10 days, 1 month, and 2 months after SBRT (*p* < 0.05 for each). In addition, the results (Figure 4) showed significant differences in TPLC and CD8^+^ T cell counts at 2 months after therapy between patients with long-term and short-term OS (*p* < 0.05 for each). However, there were no significant differences in CD19^+^ B or CD3^+^ T cells, including CD4^+^ T cells, between >2-year and <2-year survivors (*p* > 0.05).

## Discussion

Besides its direct cytotoxic effect, RT also has a systemic effect on the host mediated mainly by the immune system (25). RT shows an immunostimulatory effect via the increased release of tumor-associated antigens, high mobility group box protein, and recruitment of effector cells into the tumor microenvironment (25, 26). However, RT also has an immunosuppressive effect by increasing the expression of major histocompatibility complex class molecules, upregulating programmed death domain ligand-1 and cytotoxic T lymphocyte antigen-4, and depletion of TPLC in peripheral organs (26). The impact of the interplay between the immunostimulatory and immunosuppressive effects of SBRT on peripheral lymphocyte counts and survival should be considered.

Recently, Byun et al. (11) reported that acute severe RIL was associated with poor OS in patients with HCC. They found that RIL peaked at 1 month after therapy and partially recovered after 2 months. However, during a full year of observation, TPLC remained persistently low and did not recover to its baseline level. Our results showed that lymphopenia peaked at 10 days after SBRT and gradually recovered at 2 months, consistent with the findings of Byun et al. However, in our study, TPLC had recovered to the baseline level at the end of follow-up at 1 year. This discrepancy may be because the patients in our study all received SBRT compared to 90.2% of conventional fractionated RT (CRT) in the study by Byun et al. Unlike CRT, SBRT delivers high doses of radiation in a small number of fractions to the liver cancer while minimizing radiation exposure to the surrounding tissues. A mathematical computation model (27) detected that as the number of fractions increase, the percentage of blood receiving ≥0.5 Gy increases rapidly. The modeling determined that, while a single radiation fraction delivered 0.5 Gy to 5% of circulating cells, after 30 fractions, 99% of circulating blood had received ≥0.5 Gy. Wild (20) also reported that SBRT is associated with less severe lymphopenia than CRT in unresectable pancreatic cancer, suggesting that radiation technique affects lymphopenia. Hence, patients receiving SBRT in the present study experienced less severe lymphocyte depletion and thus may more rapidly recover to their normal values.

In addition, multivariate analysis in our study revealed that variables including TNF-α <5.5 ng/mL and post-treatment TPLC <0.45 × 10^9^/L were independent adverse factors of OS. A possible explanation for the association between TPLC and survival is that SBRT-related lymphopenia may cause immune suppression in HCC patients. It has long been thought that the tumor-infiltrating lymphocytes play a significant role in controlling cancer development and progression (28) and are associated with improved survival in various tumors (29–35). Given that circulating lymphocytes are the cells that eventually infiltrate tumors, RIL in peripheral blood might be associated with a weaker anti-tumor immune response and inferior prognosis. Another possibility is that RIL is a surrogate representing the overall patient health rather than a direct cause of decreased OS. As for TNF-α, it is one of the most important cytokines that can induce tumor necrosis without significant toxicity to normal cells. A recent study by Zhang (36) reported a positive association between TNF-α in the T1N0M0 microenvironment with the prognosis of HCC patients. This may partially explain why peripheral TNF-α level was significantly indicative of OS in the present study.

What's more, some lymphocyte subsets can be more sensitive to radiation. Recent *in vitro* studies by Falcke et al. (37) showed that NK and B cells were more radio-sensitive than T cells to cell death induction. Twenty-four hours after irradiation, NK and B cells showed decreases in viability by 10–15% and decreased by 70% at 72 h. Our results confirmed the marked depletion of B cells, with a mean B cell count that dropped to only 24% of its baseline value after SBRT and recovered more slowly than other subpopulation types. However, CD3^+^, CD4^+^, and CD8^+^ T cells as well as NK cell counts decreased by nearly half after SBRT, a less extreme decline compared to that of B cells. Regarding the discrepancy in NK cell depletion, some theories may explain our findings. First, the study by Falcke et al. focused on *in vitro* experiments which might differ from the complex human circulating system. This was proven by the study by Gustafson et al. (14) that assessed the responses of 110 immunophenotypes in the peripheral blood to SBRT in five HCC patients. They found that immature CD56^br^CD16^−^ NK cells declined dramatically after SBRT but that mature CD56^+^CD16^+^ NK cells did not (14). A study on immune responses following SBRT for Stage I NSCLC patients also indicated that B cells decreased profoundly rather than NK cells (12).

To assess the association between lymphocyte levels after SBRT with oncological outcome in HCC, we first examined their relationship in univariate analysis. The results showed that, except for B cells, other lymphocyte subsets were significantly associated with patient survival (*p* < 0.05). Stratified analysis was then performed according to survival at 2-years since SBRT. The counts of peripheral NK cells in patients with better survival remained significantly elevated compared to those in short-term survivors at 10 days to 2 months after SBRT (*p* < 0.05). NK cells, which are innate lymphoid cells with natural cytotoxicity and regulatory functions, play an integral role in the immune defense mechanism against malignant tumors such as HCC (38, 39). NK cells account for 25–50% of the total number of liver lymphocytes, suggesting their important role in liver immunity (40). The number of NK cells in both blood and tumor tissues of HCC patients is positively correlated with patient survival (39). Hence, these findings rationalized the significant association between SBRT-induced NK cell loss and inferior survival in HCC patients. In addition, the results of the present study demonstrated significant differences in TPLC and CD8^+^ T cell counts between patients with long-term and short-term OS at 2 months after SBRT (*p* < 0.05). Evidence has shown that a decreased peripheral CD8^+^ T cell count might be a risk factor indicating tumor development and metastasis and could, therefore, be associated with poor prognosis in patients with HCC (41). A meta-analysis of a large number of studies concluded that a higher CD8^+^ tumor-infiltrating lymphocyte count was also associated with higher OS in patients with HCC (42). Moreover, since our results indicated that CLP counts had usually recovered at 2 months after SBRT, the significant differences between TPLC, CD8^+^ T cell, and NK cell values at 2 months between long-term and short-term survivors may indicate that a lack of recovery might be linked to poor survival. In addition, a large PTV was independently associated with an increased risk of lymphopenia in the present study, which was confirmed by previous studies of glioblastoma (27, 43) and HCC (11).

However, the findings of this study should be carefully interpreted due to the small number of patients and the retrospective design. The population of patients with HCC that undergo SBRT might be more heterogeneous than that in our study sample; thus, there was the potential for selection bias. Another potential limitation of our study is that the blood lymphocyte counts might have been influenced by infection or cirrhosis-associated hypersplenia before HCC treatment. Furthermore, the treatments that patients received prior to SBRT were not uniform; some had previously received TACE, RFA, PEI, or surgical resection, which might have biased the results.

In conclusion, our study is the first to investigate the impact of SBRT on different CLPs in a 1-year follow-up and to explore their values for the prediction of survival in patients with HCC. Further large-scale validation studies are needed to confirm the effectiveness of lymphocyte populations in patients with HCC who receive SBRT and other RT modalities.

## Data Availability

The datasets analyzed in this manuscript are not publicly available. Requests to access the datasets should be directed to zhuangyuan.89@163.com.

## Ethics Statement

All patients have given their written informed consent. The study protocol was approved by the ethics board of Zhongshan Hospital, Fudan University (B2018-272).

## Author Contributions

YC and ZZ designed the study. YZ, BY, GC, XZ, YH, and WZ contributed to the data collection. YZ and BY analyzed the data and wrote the manuscript. All authors approved the version of the manuscript to be published.

### Conflict of Interest Statement

The authors declare that the research was conducted in the absence of any commercial or financial relationships that could be construed as a potential conflict of interest.
